# Editorial: New Approaches to Breast Cancer Radiotherapy

**DOI:** 10.3389/fonc.2021.645615

**Published:** 2021-02-24

**Authors:** Geraldine Meerbott Jacobson, Cristiane Takita

**Affiliations:** ^1^ Department of Radiation Oncology, WVU Cancer Institute, West Virginia University, Morgantown, WV, United States; ^2^ Sylvester Comprehensive Cancer Center, University of Miami Health System, Miami, FL, United States

**Keywords:** neoadjuvant radiation therapy, partial breast irradiation, MR-guided radiotherapy, boost radiation, stereotactic body radiotherapy, oligometastases, distant metastases

Multiple randomized clinical trials have confirmed the efficacy of radiation therapy in reducing local recurrence of breast cancer (1). For decades the predominant approach was six weeks of whole breast radiotherapy followed by a boost. This “one size fits all” paradigm has been questioned based on the identification of molecular markers, genomic profiling, and other prognostic factors that indicate recurrence risk for individual patients.

Breast cancer survival has improved in developed countries with almost 80% of patients surviving at least 10 years. Long-term survival reveals the late toxicity of all treatment modalities and provides an incentive to develop effective treatments that maintain quality of life. In the field of radiation oncology, we have the opportunity to tailor our treatments for each patient to improve progression free survival, minimize normal tissue toxicity and functional impairment, and respect our patient’s resources and time constraints.

We have seen a de-escalation of the surgical approach to breast cancer and the development of a more personalized targeted approach to the selection of systemic therapy. Technical innovations in radiotherapy delivery provide the opportunity to treat patients with greater precision and fewer treatments.

Radiation delivery can be modified by altering volume, dose, timing, number and overall duration of treatment consistent with optimal medical outcome and quality of life. Hypofractionation, which reduces the number and overall treatment duration, has become the recommended approach to whole breast RT (2, 3). The concept of APBI (accelerated partial breast irradiation) which minimizes volume, treatment number and duration, has been demonstrated to be appropriate for low-risk patients (4–7). Clinical trials have explored more efficient fractionation for whole breast RT (8, 9) and novel approaches to APBI. Technical progress in radiation image guidance, planning, and treatment delivery has fostered the development of SBRT (stereotactic body radiotherapy) and stereotactic ablative radiotherapy (SABR) as an ablative treatment for primary and metastatic cancer.

In this Research Topic, New Approaches to Breast Cancer Radiotherapy, we have included original research and review articles that describe SBRT for primary and metastatic breast cancer, MR-guided RT for neoadjuvant local treatment, aggressive local management of breast cancer with synchronous metastases, and a new look at the breast boost.

The article by Lee et al. describes the first experience in Korea of stereotactic partial breast irradiation. While accelerated partial breast irradiation (APBI) has been demonstrated in randomized trials to be non-inferior to whole breast radiation in selected patients with low-risk tumors, the technique has been rarely used in Korea. Reasons include the younger age distribution of Korean breast cancer patients and smaller breast volumes that limit many APBI techniques. This article demonstrates the safety and feasibility of fractionated SBRT using Cyber knife with robotic tracking and implanted fiducials. This technique offers a non-invasive APBI modality that may be well suited to small volume breasts.

The concept of oligometastatic disease proposed in the 1990’s, is that a limited number of metastases (≤5) may be amenable to ablative treatments resulting in prolonged disease-free interval or improved survival. SBRT and SABR can provide a non-invasive ablative treatment for oligometastases. The randomized phase II SABR-COMET trial demonstrated an improvement in overall survival in the SABR arm for multiple disease site, of which 20% were breast cancer (10). The role of SBRT/SABR for oligometastatic breast cancer has not been well defined. This may change with the results of NRG-BR002, an ongoing randomized study of ablative therapy for oligometastatic breast cancer. The Weykamp et al. article is a single institutional review of extracranial SBRT for oligometastatic or oligoprogressive breast cancer, which evaluates outcome and prognostic factors in this cohort of patients. As such, it contributes to the growing body of literature on the role of ablative treatment for oligometastases in breast cancer.

The rising number of breast cancer cases and the decrease in mortality from improvements in breast cancer treatment have resulted in a growing number of breast cancer survivors that experience late treatment-related toxicity. Despite the advances in radiotherapy treatment duration and oncologic outcomes with APBI (4–6), improvements in late toxicity are still needed (6, 9). Few single institutional studies have evaluated the use of neoadjuvant partial breast irradiation (PBI) treating smaller target volumes compared to adjuvant PBI, potentially reducing RT-related toxicity and improving quality of life (11–13). The evolution of Magnetic Resonance (MR)-guided RT systems has provided significant improvement in image-guided RT, with better target and normal tissue visualization. In this review paper, Groot Koerkamp et al. discuss MR-guided RT to deliver neoadjuvant PBI, outlining the steps from breast treatment planning, contouring and treatment delivery, including optimization for the use of this technique and workflow for clinical implementation.

The benefit in local control by adding a boost dose after whole breast radiotherapy has been studied in randomized trials (14, 15). However, higher radiation dose is also associated with worse cosmesis and higher cost related to additional treatment. Several guidelines have been proposed to delineate who should receive a boost, including younger patients, high grade tumors, and positive surgical margin. In this review paper, Gulstene and Raziee discuss the lack of consensus guidelines for the use of boost after hypofractionated whole breast RT and in close surgical margins, two common clinical scenarios. The authors discuss the trend in lower rates of utilization of boost after hypofractionated RT compared to conventional treatment, including the data of similar cosmetic outcomes when boost is used independent of fractionation of whole breast. The management of patients with close surgical margin has changed since ASTRO-SSO consensus guidelines recommendation of re-excision only for positive margins, resulting in significant practice variations in regard to boost for close surgical margins. The authors recommend future prospective studies to address these questions.

About 6% of newly diagnosed breast cancer patients present with Stage IV disease and an intact primary tumor. Improvements in systemic therapies including chemotherapy, HER2-target therapy, and immunotherapy have improved prognosis of this small subset of patients. The use of locoregional therapy, including surgery and/or radiotherapy has been controversial, after the results of four randomized trials showing no benefit in survival with addition of locoregional therapy (16–19). Lian et al. investigate the effect of local therapy on survival in this population using SEER database. The authors noted a decrease in the number of patients receiving surgery alone and an increase in radiotherapy alone over time. Local therapy was an independent prognostic factor for breast cancer-specific survival (BCSS). Surgery combined with radiotherapy had better BCSS compared with surgery alone and radiotherapy alone. Identification of a selected group of patients with De Novo Stage IV disease that benefit of locoregional therapy is still to be defined.

## Author Contributions

All authors contributed to the article and approved the submitted version. The individual authors added separate comments for the manuscripts they edited.

## Conflict of Interest

The authors declare that the research was conducted in the absence of any commercial or financial relationships that could be construed as a potential conflict of interest.
